# A Study on the Potential Mechanism of Shujin Dingtong Recipe against Osteoarthritis Based on Network Pharmacology and Molecular Docking

**DOI:** 10.1155/2022/1873004

**Published:** 2022-11-26

**Authors:** Zhen-zhen Yuan, Zhao Yang, Si Wu

**Affiliations:** ^1^Department of Orthopedics, Tianjin Hospital, Tianjin 300211, China; ^2^Clinical College of Orthopedics, Tianjin Medical University, Tianjin 300211, China; ^3^Department of Orthopedics, First Teaching Hospital of Tianjin University of Traditional Chinese Medicine, Tianjin 300381, China; ^4^National Clinical Research Center for Chinese Medicine Acupuncture and Moxibustion, Tianjin 300381, China

## Abstract

**Background:**

With the aging of the social population, Osteoarthritis (OA) has already become a vital health and economic problem globally. Shujin Dingtong recipe (SJDTR) is an effective formula to treat OA in China. Although studies have shown that SJDTR can significantly alleviate OA symptoms, its mechanism still remains unclear.

**Purpose:**

This study is aimed at investigating the potential mechanism of SJDTR for the treatment of OA based on network pharmacology and molecular docking.

**Methods:**

Main ingredients of SJDTR were retrieved from the Traditional Chinese Medicine Systems Pharmacology (TCMSP) database. OA disease targets were obtained from the Gene Expression Omnibus (GEO) database. The overlapped targets and signaling pathways were explored using Protein-Protein Interaction (PPI) network, Gene Ontology (GO), and Kyoto Encyclopedia of Genes and Genomes (KEGG). Following this, the core targets were employed to dock with corresponding components via molecular docking in order to further explore the mechanism of SJDTR in the treatment of OA.

**Results:**

From network pharmacology, we found 100 active components of SJDTR, 31 drug and OA-related targets, 1161 GO items, and 91 signaling pathways. Based on the analysis with PPI network and molecular docking, TP53, CCNB1, and MMP-2 were selected for the core targets of SJDTR against OA. Molecular docking demonstrated that Quercetin, Baicalein, and Luteolin, had good binding with the TP53, CCNB1, and MMP-2 protein, respectively.

**Conclusion:**

To conclude, our study suggested the main ingredients of SJDTR might alleviate the progression of OA through multiple targets and pathways. Additionally, network pharmacology and molecular docking, as new approaches, were adopted for systematically exploring the potential mechanism of SJDTR for the treatment of OA.

## 1. Introduction

Osteoarthritis (OA) is one of the most common degenerative arthritis in the global population. It causes pain and joint dysfunction, mainly in the middle-aged and elderly people, which can reduce health-related quality of life for patients. With the advent of an aging population, the incidence of OA has increased to 37.4% with 60 years of age or older in the United States, generating serious social and economic burden [1–3]. Therefore, the accurate diagnosis and treatment of OA is particularly important to lower the pain and dysfunction of patients. Nonsteroidal anti-inflammatory drug administration is one of the most commonly used nonsurgical methods for the treatment of OA. It has been demonstrated to be helpful, but relieving symptoms are limited, and accompanied by multiple side effects [4, 5]. Therefore, a large number of OA patients choose complementary and alternative medicine.

Traditional Chinese Medicine (TCM) has been applied extensively to treat many diseases due to its remarkable efficacy, abundant resources, and fewer side effects. Previous researches have shown that TCM is effective in reducing the clinical symptoms by relieving pain and improving functions of joints for OA patients [6–9]. Shujin Dingtong recipe (SJDTR), a TCM formula, is widely used for the treatment of OA in China. It contains six main Chinese herbal medicines, respectively, Angelica (Dang Gui, DG), Rhubarb (Da Huang, DH), Safflower (Hong Hua, HH), Boswellia serrata (Frankincense, Ru Xiang, RX), Myrrh (Mo Yao, MY), and Drynaria (Gu Sui Bu, GSB). Studies indicated that drugs and their extracts in SJDTR exhibited substantial anti-inflammatory effect on inhibiting the development of arthritis [10–13].

Network pharmacology (NP) is a new and promising approach integrating the theory of biology, chemistry, network analysis, and traditional pharmacology. It is a novel strategy which can be used to clarify the active compounds of TCM and their related mechanisms [14, 15]. We employed the Gene Expression Omnibus (GEO) database to obtain OA-related disease targets. The GEO database stores curated gene expression datasets, and original series. In addition, molecular docking (MD) provides an efficient method for investigational drugs through the interaction between receptors and drug molecules [16]. Thus, the above two methods exert a vital role in studying the relationship between drugs in TCM and diseases, and designing new drugs [17]. Most of the previous researches only concentrated on one TCM component for treating disease. Our motivation of the current research was to comprehensively and systematically explore the potential mechanism of SJDTR against OA based on NP and MD.

## 2. Methods

### 2.1. Screening for Main Ingredients and Potential Drug Targets for SJDTR

The main ingredients of these six Chinese medicinal herbs in SJDTR were searched from the following database: Traditional Chinese Medicine Systems Pharmacology Database and Analysis Platform (TCMSP, https://tcmspw.com/tcmsp.php). TCMSP refers to a TCM platform for Chinese herbal medicines, including 500 drugs and 30,069 ingredients.

In order to obtain compounds with good absorption, distribution, metabolism, and excretion (ADME) processes for further analysis, we required that the components in SJDTR should satisfy two of the following parameters: Oral bioavailability (OB) ≥ 30%, and Drug-likeness (DL) ≥ 0.18. Next, drug targets for main ingredients in SJDTR were obtained from the TCMSP database.

### 2.2. Acquisition of Disease Targets for OA

OA-related targets were screened from the GEO database (https://www.ncbi.nlm.nih.gov/geo/). Targets of differentially expressed genes were obtained between normal body tissues and those of OA patients. The data from GSE82107 was downloaded from the GEO database, and platform: GPL570, containing 7 normal and 10 OA samples. Genes with ∣logFC | >0.5 and adjusted *p* value<0.05 were deemed statistically significant as OA. The heat map and volcano map were drawn with the application of R software 4.1.0.

### 2.3. Acquiring Overlapping Potential Targets

Overlapping potential targets were obtained through the targets of the main components of SJDTR and the potential disease targets of OA.

### 2.4. Protein-Protein Interaction (PPI)

The PPI network of the overlapping targets from drug targets and disease targets was constructed and visualized with STRING database (https://string-db.org/). In the current study, the custom value of STRING was set to 0.4.

### 2.5. Gene Ontology (GO) and Pathway Enrichment Analysis

The GO database (http://geneontology.org/) and Kyoto Encyclopedia of Genes and Genomes (KEGG) database (https://www.kegg.jp/) were performed to further investigate targets of SJDTR for treating OA using Bioconductor, an R package (version 4.1.0), *p* value<0.05 was selected. Additionally, we researched the top 10 GO enrichments and 20 KEGG pathways with higher counts.

### 2.6. Targets-Pathways Network Construction and Analysis

To further investigate the mechanism of SJDTR on OA, the Targets-Pathways Network was built using Cytoscape 3.8.2 software. In these graphical networks, the “nodes” represented the target proteins or pathways, whereas, the interactions of target pathways were denoted as edges. The core targets were obtained from the topological analysis of the PPI network with CytoNCA.

### 2.7. Molecular Docking (MD)

In this study, we further validated the compound–target association and explored their binding patterns through MD. Three active components and three target proteins were chosen for MD. The structures of the main components as the ligand were downloaded from the PubChem Database (https://pubchem.ncbi.nlm.nih.gov/). Then, the 3D structures of target proteins were obtained from the RCSB PDB database (http://www.rcsb.org/) as receptors for verification of MD. Thereafter, the Discovery studio-2019 software was employed to realize MD between the receptors and ligands, while evaluating the datasets for docking. Root-Mean-Square Deviation (RMSD) <2A was supposed to represent consistent docking conformations of compounds.

## 3. Results

### 3.1. Main Compounds and Targets of SJDTR

From the TCMSP database, (OB) ≥ 30%, and (DL) ≥ 0.18 were adopted for screening the ingredients, and 100 compounds were selected as candidate components for further analysis, including 45 in MY, 22 in HH, 18 in GSB, 16 in DH, 8 in RX, and 2 in DG. Corresponding to the bioactive components in SJDTR, a total of 347 drug targets were retrieved from the TCMSP database, 249 for HH, 208 targets for DH, 160 targets for DG, 140 for MY, 135 for GSB, and 106 for RX.

### 3.2. Identification of OA and Overlapping Genes

**Figure 1:**
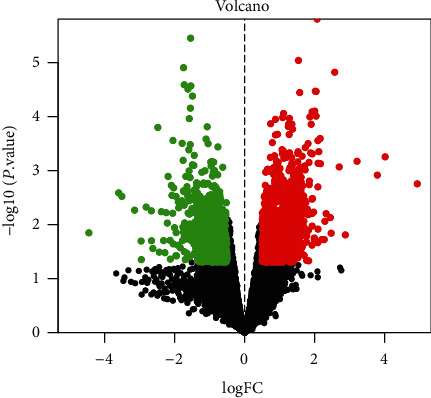
Gene volcano map from GEO database.

**Figure 2:**
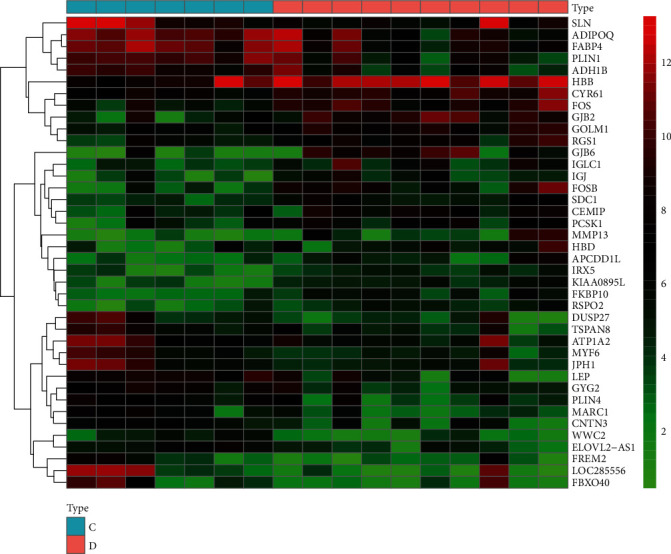
Gene hot map from GEO database. The first 7 samples were from healthy people, and the last 10 samples were from patients with OA.

**Figure 3:**
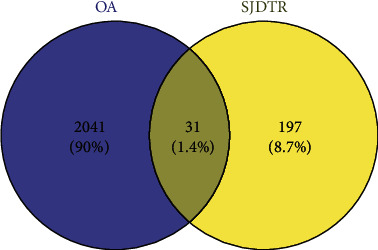
Venn diagram of the overlapping targets in SJDTR and OA.

By adopting GEO database, a total of 2072 differential genes associated with OA were screened, including 1191 upregulated genes and 881 downregulated genes with ∣logFC | >0.5 and *p* value <0.05, as displayed in Figure 1. From the volcano map, there existed a clear genetic difference between the normal group and the OA group. Red represented upregulated genes, green denoted downregulated genes, and black stood for no significant change. The hot map indicated the most significant up and downregulation of the first 40 genes, as presented in Figure 2. In the hot map, red indicated upregulated genes, green referred to downregulated genes, and black represented no significant difference. As shown in Figure 3, 31 overlapping targets were obtained from drug targets in SJDTR associating with OA, and the detailed information was revealed in supplementary table 1. It suggested that such targets might play an important role in the treatment of OA.

### 3.3. Compounds-Targets Network and Analysis

**Figure 4:**
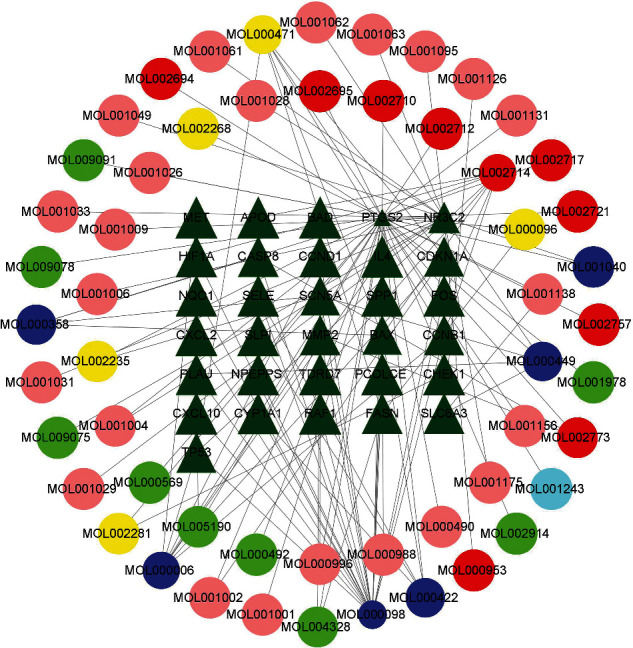
Compounds-targets network in SJDTR and OA.

SJDTR, as a TCM formulas, has numerous compounds, which may act on multiple targets and reveal cooperative effects in the treatment of OA. As the above results, 31 target genes and 54 drug components were performed to build the drug compounds and targets network, including 85 nodes and 117 edges (Figure 4). As presented in Figure 4, the nodes in the outer ring were compounds, and the green nodes in the middle were gene targets, while the edges indicated the interactions between them. As depicted in the figure, MOL000098 (Quercetin) showed the most target interactions (degree = 21), followed by MOL002714 (Baicalein, degree = 8), MOL000006 (Luteolin, degree = 8), MOL000471 (Aloe Emodin, degree = 6), and MOL000422 (Kaempferol, degree = 5).

### 3.4. PPI Network Construction

**Figure 5:**
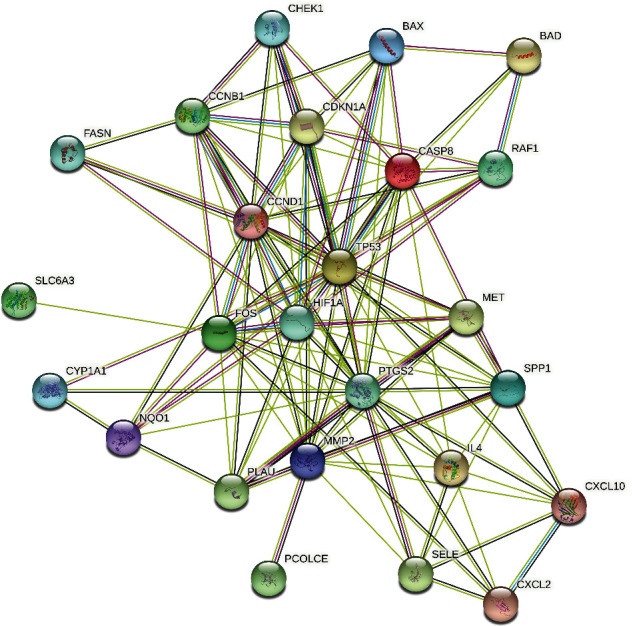
The PPI network of overlapping targets.

In the PPI network, the interaction was presented between these overlapping targets, and we imported 31 targets with a relatively close interaction to build the PPI network, consisting of 31 nodes and 119 edges (Figure 5). It showed that 25 proteins would be the focus in the current PPI network. NR3C2, NPEPPS, TDRD7, SLPI, APOD, and SCN5A were not associated with other proteins. In Figure 5, CCND1, including the most edges, could be related to the other 13 proteins, followed by CASP8 (12 edges) and FOS (11 edges).

### 3.5. Analyses of GO and KEGG Pathway Enrichment

**Figure 6:**
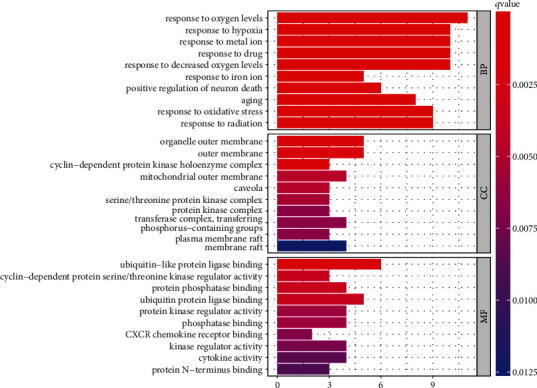
GO functional analysis of 31 intersected targets.

GO biological enrichment analysis illustrates gene function from the following three aspects: biological process (BP), cellular component (CC), and molecular function (MF). R language was used for GO analysis, and a total of 1161 GO items were recognized. There were 1115 terms in BP, 13 in CC, and 33 in MF. The top 10 terms in each category were shown in Figure 6. In terms of BP, it mainly involved oxygen levels, hypoxia, metal ion, drug, decreased oxygen levels, iron ion, neuron death, aging, oxidative stress, and radiation. In the CC, it mainly involved organelle outer membrane, outer membrane, cyclin-dependent protein kinase holoenzyme complex, and so on. In terms of MF, it mainly contained ubiquitin-like protein ligase binding, cyclin-dependent protein serine/threonine kinase regulator activity, protein phosphatase binding, etc.

**Figure 7:**
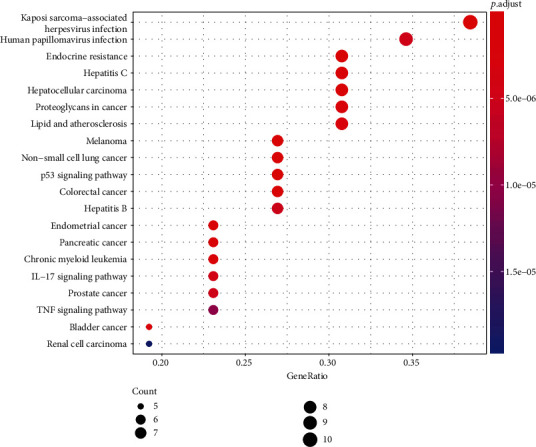
KEGG pathway analysis of 31 intersected targets.

To further characterize the underlying pathways by which SJDTR alleviated OA, KEGG pathway analysis was performed through the R software Bioconductor package. A total of 91 pathways were identified, and the top 20 pathways were screened, including P53, IL-17, and TNF signaling pathway, etc. (Figure 7). These pathways might exert a key role in the treatment of OA.

### 3.6. Proteins-Pathways Network and Analysis

**Figure 8:**
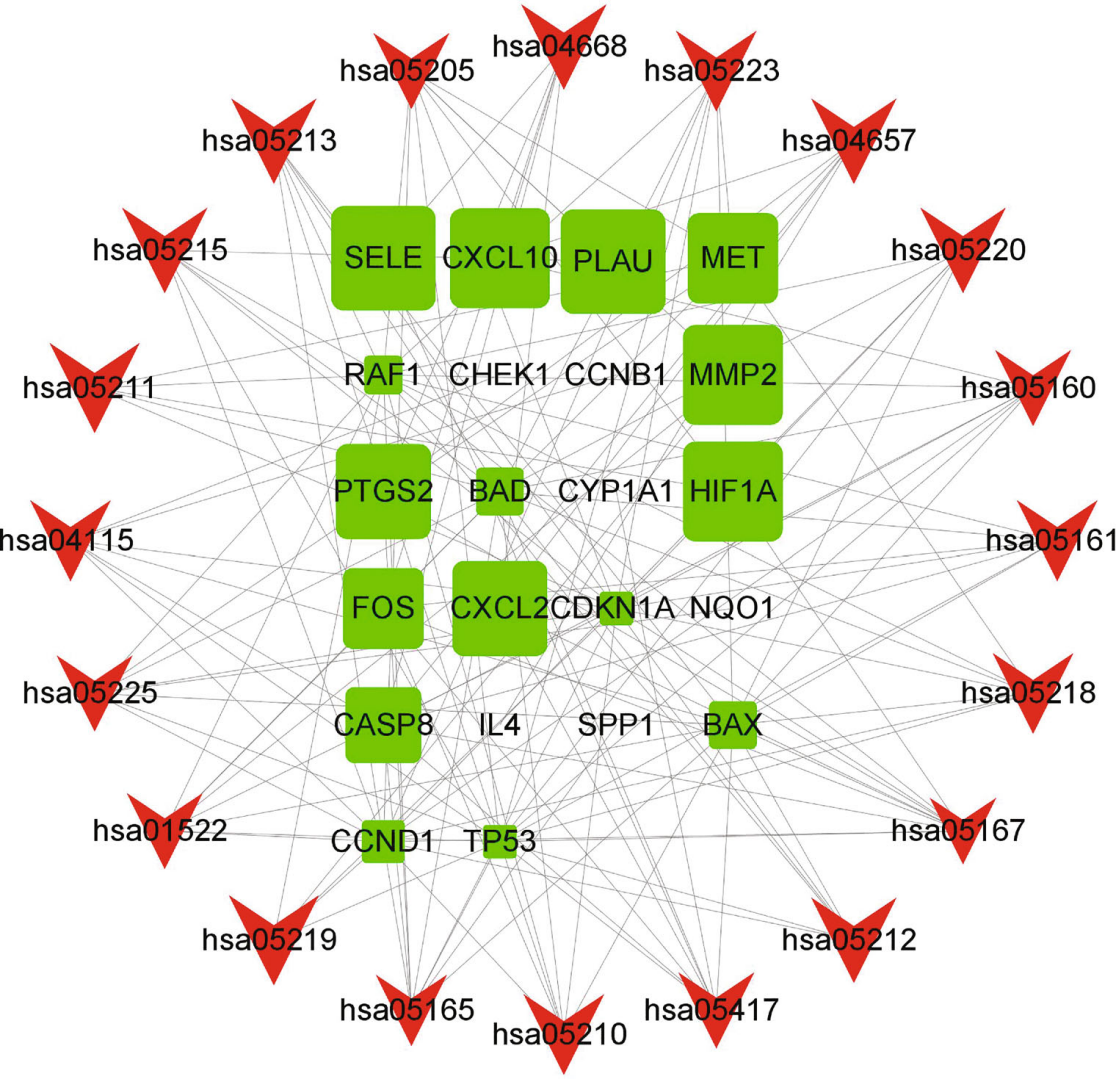
Proteins-pathways network.

**Figure 9:**
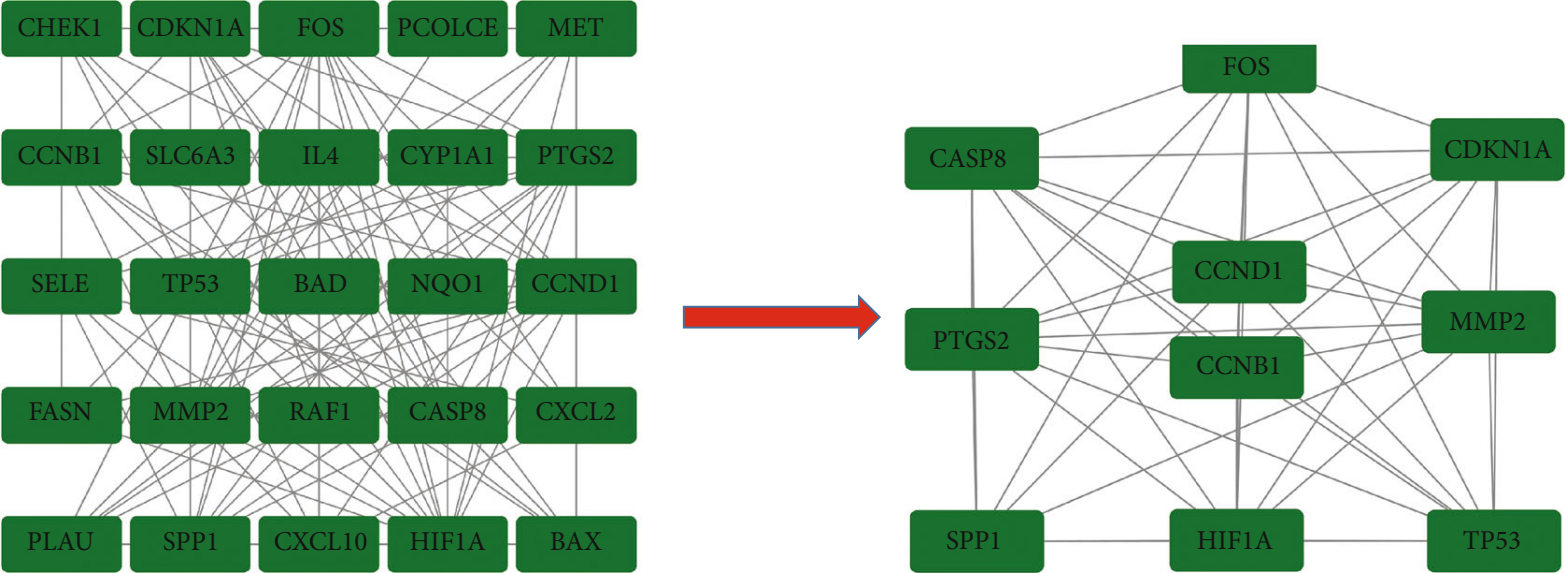
The 10 core targets by CytoNCA.

To further understand the molecular mechanism of SJDTR on OA, a targets-pathways network was built based on overlapping proteins and pathways involving most targets by adopting Cytoscape software (Figure 8). According to the figure, it included 42 nodes and 140 edges. The green nodes represented target proteins, and the red nodes in the outer circle were pathways. However, the edges connected proteins in common pathway. As shown in the figure, TP53 was interacted with 17 pathways, followed by CDKN1A and RAF1. Thereafter, 10 core targets (Figure 9) were acquired with CytoNCA analysis, detailed information was shown in supplementary table 2.

### 3.7. Results of MD

In our study, TP53, CCNB1, and MMP-2 from the core targets were selected as receptors, and three corresponding components with a higher degree were used as ligands for MD, namely, Quercetin, Baicalein, and Luteolin. Discovery studio software was adopted to perform MD. Compared with the crystal structure, the RMSD values of TP53, CCNB1, and MMP-2 complex were 0.81A, 0.58A, and 1.35A (Table 1), respectively. The conformation of the ligand in the original crystal structure of the target proteins overlapped with that of the ligand after docking (RMSD<2A).

**Figure 10:**
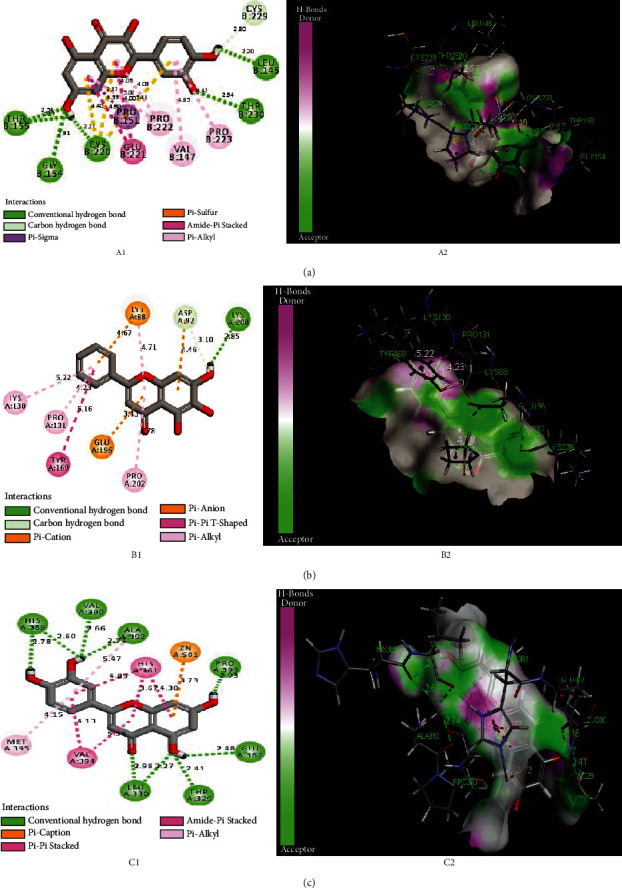
(a) TP53 and Quercetin; (b) CCNB1 and Baicalein; (c) MMP-2 and Luteolin.

The LibDockScore of TP53 with Quercetin was 138.06. The hydrogen bonding provided the major binding affinity between the receptor and the ligand. There were five hydrogen bondings of TP53 and Quercetin, including THR B:155, GLY B:154, CYS B:220, LEU B:145, and THR B:230 (Figure 10(a)). The LibDockScore of CCNB1 and Baicalein was 92.64, and the hydrogen bonding was LYS A:200 (Figure 10(b)). The LibDockScore of MMP-2 and Luteolin was 133.22. In addition, the hydrogen bondings were HIS A:389, VAL A:390, ALA A:392, PRO A:393, GLU A:362, THR A:329, and LEU A:330 (Figure 10(c)) (Table 1).

## 4. Discussion

OA is a degenerative joint disorder that is primarily characterized by destruction of the articular cartilage, cartilage matrix degradation, and synovium inflammation. The treatments for OA primarily include physical measures, drug therapy, and surgery. Currently, the research of TCM in the treatment of OA has attracted more attention due to its validity and security. This study concentrated on the mechanism of SJDTR for treating OA. SJDTR, as a classical TCM formulation, comprises six main herbs, including Angelica, Rhubarb, Safflower, Boswellia serrata, Commiphora, and Drynaria fortunei. Studies revealed that the above traditional herbs were significantly effective in the treatment of OA. Magdalou et al. reported that Angelica would inhibit cartilage damage and promote its repair to alleviate joint degeneration [10]. Hu et al. indicated that Hydroxysafflor yellow A (HSYA), the main active component in safflower, exhibited substantial anti-inflammatory effects in OA through suppressing interleukin (IL) -1*β*-induced activation of the nuclear factor-kappa B(NF-*κ*B) and Mitogen-activated protein kinases (MAPK) cascades [11]. Kulkarni et al. showed that Ru Xiang and extract significantly improved pain and reduced the level of inflammatory factors in serum of knee OA patients, including IL-2, IL-4, IL-6, TNF-*α*, and IFN-*γ* [12]. Total flavonoids of Rhizoma Drynariae exhibited chondroprotective effects via restoring the MMP/TIMP balance in OA rat models by suppressing the activation of the NF-*κ*B and PI3K/AKT pathways [13].

SJDTR, as an effective TCM for OA, has multiple components and multiple interactions among its components. As observed in Figure 4, Quercetin (MOL000098) is associated with the most overlapping genes, including the 10 core genes. Previous studies have shown that, as a member of the flavonoid family, Quercetin has a variety of biological activities, especially its anti-inflammatory and antioxidant properties in degenerative diseases. Li et al. indicated that Quercetin alleviated rat OA by inhibiting inflammation and apoptosis of articular cartilage through downregulating IRAK1/NLRP3 signaling pathway [18]. Lv et al. showed that Quercetin inhibited knee joint degeneration by reducing inflammatory factor secretion (MMP-13), regulating autophagy via TSC2-RHEB-mTOR pathway, inhibiting apoptosis as well as increasing the secretion of collagen II and aggrecan [19]. Additionally, Baicalein (MOL002714), extracted from a Chinese herbal medicine (Scutellaria baicalensis), has been extensively applied in the therapy for inflammation. Chen et al. showed that Baicalein significantly reduced MMPs expression via MAPK signaling pathway in order to attenuate OA progression [20]. Moreover, Fei et al. demonstrated that Luteolin (MOL000006) prevented cartilage destruction by reducing expressions of inflammatory factors, including NO, PGE2, TNF-*α*, COX2, and MMPs [21]. Zhou et al. indicated that Luteolin activated the AMPK and Nrf2 pathways in order to relieve joint degeneration [22]. Subsequently, Ding et al. reported that emodin (MOL000471) attenuated OA progression by suppressing the NF-*κ*B and Wnt/*β*-catenin pathways [23]. Xiao et al. implicated that Kaempferol (MOL000422) reduced the effects on inflammation by XIST/miR-130a/STAT3 pathway in chondrocytes, as well as promoted secretion of extracellular matrix (ECM) [24]. Collectively, our study indicated that the main compounds in SJDTR had various pharmacological effects, such as inhibiting inflammatory and apoptotic, as well as promoting ECM secretion for the treatment of OA.

By exploring PPI network, we found that 25 overlapping proteins had a relatively close interaction in the treatment of OA. Caspase-8 is an initiator protein to activate downstream Caspases to trigger apoptosis, with Bad and Bax playing an apoptosis-promoting role during the process of apoptosis [25]. CCNB1, CDKN1A, CCND1, and TP53 were associated with cell cycle, DNA replication and mitosis, regulating cell proliferation, aging, and apoptosis [26–28]. LI-4, CXCL-2, and-10, as proinflammatory factors may lead to the development of inappropriate inflammatory reaction after injury [29, 30]. Increasing amounts of evidence has shown that the expressions of above proteins play a great role in OA development and progression.

In this study, based on the GO and KEGG analyses, the potential targets for SJDTR in the treatment of OA were associated with P53, IL-17, NF-*κ*B, MAPK, and TNF signaling pathways. P53 protein, expressed by TP53 gene, is recognized as apoptotic protein by both inhibiting cellular proliferation and inducing cellular apoptosis. Xu et al. found that the expression of P53 protein was increased in OA progression, and the reduction of P53 could alleviate OA progression [31]. Zhu et al. reported that expression of P53 was significantly higher in knee OA patients than that in healthy controls, and with the grades of OA increasing, P53 mRNA expression were gradually upregulated [28]. IL-17 is considered an important inflammatory cytokine involved in the pathogenesis of OA. The IL-17 family is composed of six members, IL-17A to IL-17F. Zhou et al. reported that the expression of IL-17 was observed in articular cartilage cells from samples of OA patients. Elevated IL-17 was responsible for local inflammation in OA synovium by activating PI3K/AKT/mTOR pathway [32]. The NF-*κ*B signaling is another known pathway associated with the pathogenesis of OA. It was activated by proinflammatory cytokines and could modulate catabolic events of articular cartilage and extracellular matrix in OA progression [33]. The expression levels of P65, MMP-13, and IL-6 involved in NF-*κ*B signaling were increased in OA articular tissues. Chow et al. showed that the activated NF-*κ*B pathway enhanced the expressions of inflammatory factors and matrix-degrading enzymes, including IL-6 and MMPs [34]. MAPK signaling pathways were participated in multiple cellular processes, including cell proliferation, apoptosis, and survival in the cartilaginous tissues. Activated MAPK pathway might upregulate the expressions of inflammatory cytokines such as MMPs, IL-1, and TNF-*α*, while p38, p-JNK, and p-ERK were involved in this pathway. Zhou et al. indicated that Kin prevented MAPK signaling molecules p-JNK, p-ERK, and p-P38 in order to alleviate OA progression [35]. TNF is described as proinflammatory cytokines associated with the pathophysiological processes in the course of inflammatory, infectious, and malignant processes. The TNF signaling pathway also contribute to a diverse range of cellular responses, including cell proliferation, apoptosis, survival, and migration. Zhao et al. reported that the expression of TNF-*α* was elevated in OA chondrocytes, and the NF-*κ*B signaling pathway was activated and mediated by TNF-*α* protein [36].

Based on CytoNCA analysis, 10 core targets were acquired, including TP53, CCNB1, CDKN1A, MMP-2, HIF-1A, CCND1, CASP8, FOS, PTGS, and SPP1. MMPs, stimulated by inflammatory cytokines, can degrade all the components of ECM. MMP-2 (the gelatinase), as a major member of MMPs family, can degrade type IV collagen components and generate the destruction of articular cartilage [37]. Fei et al. demonstrated that expression of MMP-2 increased significantly in OA rat, and luteolin, a natural flavonoid, considerably reduced its expression in order to attenuate OA progression [21]. CCNB1 is considered as cycle protein involved in regulation of cell division. Huan et al. found that the expression of CCNB1 was upregulated in late-stage knee OA [26]. Zhou et al. indicated that platelets promoted the proliferation of osteoarthritic chondrocytes through the ERK/CDK1/cyclin B1 signaling pathway [38]. The HIF-1 family plays an important role in the progression of articular cartilage degeneration, which is involved in angiogenesis, cell proliferation, apoptosis, and viability. HIF-1A, as a main member of HIF-1, could regulate chondrocytes with the aim of adapting to a hypoxic environment [39]. Chen et al. found that there existed a significant increase in HIF-1A expression in OA tissues, and that circRNA-UBE2G1 facilitated the OA progression by regulating the miR-373/HIF-1a axis [40]. Caspase-8 is widely accepted as an apical apoptotic factor involved in cell death pathways including intrinsic and extrinsic apoptosis that is critically vital for the progression of OA [41]. Ni et al. discovered that Pravastatin significantly reduced high expression of Caspase-8 in articular cartilage in order to reverse apoptosis of chondrocytes in OA rats [42].

Through MD analysis and verification, the major components in SJDTR, Quercetin, Baicalein, and Luteolin, had good binding with TP53, CCNB1, and MMP-2 from the core targets protein. Moreover, our data may provide evidence that the main components are reliable and associated with the targets for OA.

## 5. Conclusion

In summary, the pharmacological mechanism of the effects of SJDTR on OA was investigated with the combination of NP and MD. Our results suggested that SJDTR might alleviate the progression of OA mainly via the regulation of cell cycle, proliferation, apoptosis, and inflammation. Nevertheless, this study was only a theoretical prediction. Moreover, further clinical studies and basic experiments should be undertaken to clarify these findings.

## Figures and Tables

**Table 1:** Results of MD.

Protein	RMSD(A)	LibDockScore
TP53	0.81	138.06 (quercetin)
CCNB1	0.58	92.64 (Baicalein)
MMP2	1.35	133.22 (Luteolin)

## Data Availability

The figures and tables used to support the findings of this study are included within the article, and the original data are available from the first author or corresponding author upon request.

## Supplementary Materials

Supplementary Materials Supplementary table 1: overlapping targets in SJDTR and OA. Supplementary table 2: information on 10 core targets.
