# Supplementary material for: A Study on the Potential Mechanism of Shujin Dingtong Recipe against Osteoarthritis Based on Network Pharmacology and Molecular Docking
Source: Comput Math Methods Med. 2022 Nov 26;2022:1873004. doi: 10.1155/2022/1873004 (PMC9719423; doi:10.1155/2022/1873004)
Supplement: Supplementary Materials — Supplementary table 1: overlapping targets in SJDTR and OA. Supplementary table 2: information on 10 core targets. [file 1873004.f1.zip › Supplementary table 1.docx]

### Supplementary table 1：Overlapping targets in SJDTR and OA

| **UniProt ID** | **Protein** | **Gene symbol** |
| --- | --- | --- |
| P35354 | Prostaglandin G/H synthase 2 | PTGS2 |
| [Q14524](https://www.uniprot.org/uniprot/Q14524) | Sodium channel protein type 5 subunit alpha | SCN5A |
| [O14757](https://www.uniprot.org/uniprot/O14757) | Serine/threonine-protein kinase Chk1 | CHEK1 |
| [Q07812](https://www.uniprot.org/uniprot/Q07812) | Apoptosis regulator BAX | BAX |
| [Q14790](https://www.uniprot.org/uniprot/Q14790) | Caspase-8 | CASP8 |
| [P38936](https://www.uniprot.org/uniprot/P38936) | Cyclin-dependent kinase inhibitor 1 | CDKN1A |
| [P04637](https://www.uniprot.org/uniprot/P04637) | Cellular tumor antigen P53 | TP53 |
| [P49327](https://www.uniprot.org/uniprot/P49327) | Fatty acid synthase | FASN |
| [P14635](https://www.uniprot.org/uniprot/P14635) | G2/mitotic-specific cyclin-B1 | CCNB1 |
| [P08235](https://www.uniprot.org/uniprot/P08235) | Mineralocorticoid receptor | NR3C2 |
| [Q01959](https://www.uniprot.org/uniprot/Q01959) | Sodium-dependent dopamine transporter | SLC6A3 |
| [P00749](https://www.uniprot.org/uniprot/P00749) | Urokinase-type plasminogen activator | PLAU |
| [P04798](https://www.uniprot.org/uniprot/P04798) | Cytochrome P450 1A1 | CYP1A1 |
| [P16581](https://www.uniprot.org/uniprot/P16581) | E-selectin | SELE |
| [P03973](https://www.uniprot.org/uniprot/P03973) | Antileukoproteinase | SLPI |
| [Q92934](https://www.uniprot.org/uniprot/Q92934) | Bcl2-associated agonist of cell death | BAD |
| [P15559](https://www.uniprot.org/uniprot/P15559) | NAD(P)H dehydrogenase [quinone] 1 | NQO1 |
| [P24385](https://www.uniprot.org/uniprot/P24385) | 1/S-specific cyclin-D1 | CCND1 |
| [P08253](https://www.uniprot.org/uniprot/P08253) | 72 kDa type IV collagenase | MMP-2 |
| [P05112](https://www.uniprot.org/uniprot/P05112) | Interleukin-4 | IL-4 |
| [P08581](https://www.uniprot.org/uniprot/P08581) | Hepatocyte growth factor receptor | MET |
| [P01100](https://www.uniprot.org/uniprot/P01100) | Proto-oncogene c-Fos | FOS |
| [Q16665](https://www.uniprot.org/uniprot/Q16665) | Hypoxia-inducible factor 1-alpha | HIF-1A |
| [Q8NHU6](https://www.uniprot.org/uniprot/Q8NHU6) | Tudor domain-containing protein 7 | TDRD7 |
| [P05090](https://www.uniprot.org/uniprot/P05090) | Apolipoprotein D | APOD |
| [P04049](https://www.uniprot.org/uniprot/P04049) | RAF proto-oncogene serine/threonine- protein kinase | RAF1 |
| [P19875](https://www.uniprot.org/uniprot/P19875) | C-X-C motif chemokine 2 | CXCL2 |
| [P02778](https://www.uniprot.org/uniprot/P02778) | C-X-C motif chemokine 10 | CXCL10 |
| [P10451](https://www.uniprot.org/uniprot/P10451) | Osteopontin | SPP1 |
| [Q15113](https://www.uniprot.org/uniprot/Q15113) | Procollagen C-endopeptidase enhancer 1 | PCOLCE |
| [P55786](https://www.uniprot.org/uniprot/P55786) | Puromycin-sensitive aminopeptidase | NPEPPS |
