# Supplementary material for: A Study on the Potential Mechanism of Shujin Dingtong Recipe against Osteoarthritis Based on Network Pharmacology and Molecular Docking
Source: Comput Math Methods Med. 2022 Nov 26;2022:1873004. doi: 10.1155/2022/1873004 (PMC9719423; doi:10.1155/2022/1873004)
Supplement: Supplementary Materials — Supplementary table 1: overlapping targets in SJDTR and OA. Supplementary table 2: information on 10 core targets. [file 1873004.f1.zip › Supplementary table 2.docx]

### Supplementary table 2： Information on 10 core targets

| **UiProt ID** | **Target genes** | **Protein** | **Degree** | **Compounds** |
| --- | --- | --- | --- | --- |
| [Q14790](https://www.uniprot.org/uniprot/Q14790) | CASP8 | Caspase-8 | 9 | MOL000358,MOL002773  MOL000098 |
| [P24385](https://www.uniprot.org/uniprot/P24385) | CCND1 | 1/S-specific cyclin-D1 | 9 | MOL000006,MOL000098 |
| [P01100](https://www.uniprot.org/uniprot/P01100) | FOS | Proto-oncogene c-Fos | 9 | MOL002714,MOL000098 |
| [Q16665](https://www.uniprot.org/uniprot/Q16665) | HIF-1A | Hypoxia-inducible factor 1-alpha | 9 | MOL002714,MOL000098 |
| [P08253](https://www.uniprot.org/uniprot/P08253) | MMP-2 | 72 kDa type IV collagenase | 9 | MOL001002,MOL000006  MOL002773,MOL000098 |
| P35354 | PTGS2 | Prostaglandin G/H synthase 2 | 9 | MOL001004,MOL001026  MOL001131,MOL001138  MOL001156,MOL000490  MOL000988,MOL000996  MOL001243,MOL002235  MOL002268,MOL002281  MOL000358,MOL000471  MOL000096,MOL000449  MOL001040,MOL001978  MOL002914,MOL000422  MOL004328,MOL000492  MOL005190,MOL000569  MOL000006,MOL009078  MOL009091,MOL002694  MOL002695,MOL002710  MOL002712,MOL002714  MOL002717,MOL002721  MOL002757,MOL002773  MOL000098,MOL001001 |
| [P04637](https://www.uniprot.org/uniprot/P04637) | TP53 | Cellular tumor antigen P53 | 9 | MOL000098,MOL000471  MOL000006,MOL002714 |
| [P14635](https://www.uniprot.org/uniprot/P14635) | CCNB1 | G2/mitotic-specific cyclin-B1 | 8 | MOL000471,MOL000006  MOL002714,MOL000098 |
| [P38936](https://www.uniprot.org/uniprot/P38936) | CDKN1A | Cyclin-dependent kinase inhibitor 1 | 8 | MOL001002,MOL000471  MOL000006,MOL000098 |
| [P10451](https://www.uniprot.org/uniprot/P10451) | SPP1 | Osteopontin | 7 | MOL000098 |
